# The health of Arab-Americans living in the United States: a systematic review of the literature

**DOI:** 10.1186/1471-2458-9-272

**Published:** 2009-07-30

**Authors:** Abdulrahman M El-Sayed, Sandro Galea

**Affiliations:** 1 Center for Social Epidemiology and Population Health, Department of Epidemiology, University of Michigan School of Public Health, Ann Arbor, MI, USA; 2 University of Michigan Medical School, Ann Arbor, MI, USA; 3 Survey Research Center, Institute for Social Research, University of Michigan, Ann Arbor, MI, USA; 4 Center for Global Health, University of Michigan, Ann Arbor, MI, USA

## Abstract

**Background:**

Despite substantial attention paid to Arab-Americans (AAs) in the media and in public discourse, there is limited research about the health of AAs in the United States (US) in the public health literature. This review aims to synthesize the extant peer-reviewed literature concerned with the health of AAs living in the US.

**Methods:**

We summarize existing research on the prevalence, relative burden compared to other ethnic and racial groups, and determinants of diseases within each morbidity cluster among AAs living in the US.

**Results:**

Available evidence suggests that the health of AAs may differ from that of other ethnic and racial groups in the US, and that exposures specific to this ethnic group, such as immigration, acculturation, and discrimination may be important in the etiology of several diseases among AAs.

**Conclusion:**

Given the growth of this ethnic group and its marginalization in the current sociopolitical climate, more research about the health of AAs in the US seems warranted. We summarize relevant methodological concerns and suggest avenues for future research.

## Background

Arab-Americans (AAs) are residents of the United States (US) who trace their ancestral, cultural, or linguistic heritage or identity back to one of 22 Arab countries. Many recent high profile events–including the ongoing Arab-Israeli conflict, the September 11, 2001 attacks, and the war in Iraq–have put this group under increasing scrutiny in the popular press [1,2]. Despite this greater attention to AAs in the media and in public discourse in the US, there is limited research about the health and well-being of AAs in the US in the epidemiologic and public health literature.

There are many reasons why health indicators among AAs may be different than those in the general population at large. First, AAs are disproportionately recent immigrants to the US [3]. Second, they share a set of cultural norms, heavily influenced by Islamic behavioral restrictions, that may substantially influence health behaviors. Third, this group has, in the past few decades, been marginalized from the general population, and increasingly so in the past several years [4,5].

The aim of this review was to systematically assess the peer-reviewed literature concerned with the health of AAs living in the US and to summarize the prevalence and correlates of key health indicators among AAs. We considered the peer-reviewed epidemiologic and public health literature about AA health since 1980. In so doing, we aimed to synthesize current knowledge about the health of AAs, inform practitioners to particular health risks within the Arab-American (AA) community, and encourage research that can address unanswered questions about the health of this ethnic group.

### Arab-Americans in the US

Arab immigration to the US occurred in three waves. The first began in 1875 and was comprised largely of Syrian and Lebanese Christian immigrants, most of whom were uneducated and worked unskilled jobs in the US. Following World War I, the Quota and Johnson-Reed Acts of 1924 limited the number of Arab immigrants allowed entry into the US each year, bringing to a close the first generation of Arab immigration.

Post World War II pro-immigration policy ushered in the second wave of Arab immigrants. The demographic characteristics of Arab immigrants during this wave differed substantially from the first: the majority came to the US fleeing post-war political upheavals in Egypt, Syria, Iraq, and Palestine. In contrast to those who immigrated during the first wave, this group was better educated, spoke fluent English, and worked white-collar jobs in the US.

The third wave of Arab immigration to the US began in 1965 after the Immigration Act of that year ended the quota system favoring European immigrants. This generation was the best educated compared to Arab immigrants during the other two waves [6-11]. The 1990s saw an immense increase in the number of Arab immigrants to the US: it is estimated that the AA population grew by 65% between 1990 and 2000 [3]. Most recent estimates of the AA population in the US range from 1.2 million-3.5 million [3,12].

Approximately 94% of AAs in the US live in metropolitan areas, which is substantially higher than the 80% of the general US population living in metropolitan areas [3]. Michigan is the state with the highest concentration of AAs of any US state (at about 1%) and, in particular, the largest urban concentration of AAs in the US lives in metropolitan Detroit, MI, which is home to approximately 400,000 AAs. California, with approximately 715,000 AAs, is the state with the largest absolute population of AAs in the US [12].

As compared to the general US population, AAs are on the whole better educated, more affluent, and more likely to be entrepreneurs or own businesses [3]. The largest current proportion of AAs in the US is Roman Catholic (35%), followed by Muslim (24%), and then Eastern Orthodox Christian (18%). Thirty-nine percent of AAs in the US are of Lebanese origin, while Egyptian-Americans and Syrian-Americans comprise 12% each. More than 80% of AAs in the US are American citizens [3].

## Methods

This review assesses current knowledge about the health of AAs and will encompass the peer-reviewed literature published between 1980 and 2008. We limited our review to these years in order to reflect current thinking about the relation between ethnicity and health. The literature reviewed was identified through the MEDLINE, Web of Science, BIOSYS previews, and Current Content Connect databases using the Web of Knowledge interface, and it covered empirical studies about any aspect of AA health in the US. All queries were carried out by the primary author during the month of July, 2008. A detailed account of the search strategy and results is provided in the additional files 1 &2.

We restricted our review to those studies that empirically and specifically assessed the health of AAs living in the US. The search was limited to English-language studies. Keywords and terms used for the search included the following: Arab, America*, Middle*Eastern, Chaldean, Diabet*, glucose, metaboli*, "heart disease", infarct*, heart, MI, CVD, atherosclero*, stroke, ischemi*, cancer, neoplas*, tumor, leukemia, lymphoma, depress*, anxiety, PTSD, post*trauma*, substance, mood, schizophren*, mental, psych, matern*, pregnan*, ped*, preterm, birth, abuse, tobacco, smok*, water*pipe. We also assessed the references of all papers reviewed to identify any papers that were not detected using the systematic searches.

## Results

The original search provided 88 publications of which 54 were removed because they were either meeting abstracts or were not empiric in design (i.e., commentaries or discussion pieces). This review, therefore, covers 34 studies, which are reviewed in detail in Table S1, included in the additional files [additional file 2]. There were no papers referenced in any of these papers that were not detected by our systematic review. We organized our findings around eight morbidity clusters: diabetes, cardiovascular disease (CVD), tobacco use, psychological well-being, cancer, women and children's health, health and illness psychology, and miscellaneous reports. The studies reviewed here assess the health of AAs in the US using two empirical study designs: 27 of the studies were cross-sectional analyses and the remaining 7 were retrospective cohort analyses or medical chart reviews. We found no articles published about the health of AAs that used a prospective cohort design. Also, 26 of the articles that we found concerning the health of AAs were carried out in Michigan. Of those, 24 took place in metropolitan Detroit. Of the reviewed articles, only 7 reported direct comparisons of health metrics between AAs and other racial/ethnic groups. Throughout this section, we will first summarize point estimates of health indicators where available, highlighting differences between point estimates for AAs compared to other groups in the US population, and then we will discuss measures of association assessing determinants of each health indicator among AAs in the US.

### Cardiovascular Disease

There is little consensus in the literature about the burden of cardiovascular disease (CVD) and its risk factors among AAs relative to other groups. The prevalence of hypertension among AAs is between 13–20% [13-15]. The prevalence of hypertension among AAs is comparable to that among non-Hispanic whites (23–25%) [14,16], higher than among African-Americans (8%) in a comparative study [15], but lower than among African-Americans (32%) according to data from the third National Health and Nutrition Examination Survey (NHANES III) [16]. The prevalence of other risk factors for CVD, including overweight, is higher among AAs as compared to the general US population [17].

Two studies have assessed the determinants of CVD risk factors among AAs. In one study, elevated body mass index (BMI) was associated with elevated blood pressure, increased blood glucose, increased total cholesterol and decreased high-density lipoprotein (HDL)-cholesterol among AAs [17]. Length of stay in the US was positively associated with risk for hypertension in another study [14].

### Tobacco Use

Two studies assessed the prevalence of tobacco use among AA adults. In one study, tobacco use was found to be more prevalent among AA adults than among the general US adult population. In a randomly selected sample of 237 AA adults in Michigan, 39% of respondents were current smokers, as compared to 29% both in Michigan and nationally [18]. Eleven percent of respondents were former smokers, as compared to 23% in Michigan and 26% nationally [18,19]. This study concluded that AAs had a higher smoking rate, a lower quitting rate, and a much lower quit ratio when compared with national and state data. Determinants of smoking in this sample were young age and male gender [20]. Another study found that the prevalence of smoking during pregnancy among AAs (6%) was lower than that published among non-Hispanic whites (20%) and African-Americans (13%) [21,22].

Several studies have assessed the prevalence and determinants of tobacco use among AA adolescents. The prevalence of ever smoking a cigarette among AA teens was between 29–45% across several studies, while the prevalence of one-month history of smoking was between 7–17% in the extant studies about the prevalence of adolescent tobacco use among AAs [23-25]. Rates of ever smoking (50%) and one-month history of smoking (22%) were higher among the general population of US adolescents than among AAs [25-27]. Water-pipe (a traditional Arab pipe used to smoke tobacco) smoking was more prevalent among AA youth than among the general adolescent population [25,28].

There are several risk factors for tobacco use among AA adolescents that have been identified; these include male gender, peer-smokers, lack of religious influence, lack of perceived negative consequences of smoking, poor grades, high stress, family members who smoke, reporting many hours of smoking exposure daily, receiving offers to smoke, tobacco-related advertisements and mail, belief that tobacco can help make friends, and having a US-born mother [23-25]. Among the general population, determinants of adolescent smoking include parental socioeconomic status, personal income, parental smoking, parental attitudes, sibling smoking, peer smoking, peer attitudes and norms of smoking, family environment, attachment to family and friends, school factors, risk behaviors, lifestyle, stress, and depression/distress [29]. The only determinant of water-pipe smoking found among a sample of 297 AA adolescents of Yemeni descent was experimental tobacco use, which itself was significantly correlated with age and peer smoking influence [30]. Predictors of water-pipe use in the general population in this study included cigarette use, marijuana use, and male gender [28].

### Diabetes

Several studies have assessed the prevalence and determinants of diabetes and related endocrine diseases among AAs in the US. However, there is little consensus in the literature about the burden of diabetes among AAs relative to the general US population. In cross-sectional analyses using convenience samples in metropolitan Detroit, the prevalence of diabetes among AAs was shown to be between 16–33% [15,31,32]. This suggests that the prevalence of diabetes among AAs is greater than among non-Hispanic whites (9%), African-Americans (10%), and Hispanics (11%), when compared with data from NHANES III [33,34]. By contrast, in a national study using data from the National Health Interview Survey (NHIS) 2000–2003, the prevalence of diabetes among persons born in the Arab world was 5% as compared to 7% among non-Hispanic whites [14]. In a third, comparative study of African-American and AA women in Michigan, there was no significant difference between the prevalence of diabetes among AA women (28%) and African-American women (22%) [15].

One study considered the prevalence of metabolic syndrome among AAs, which was comparable to the general US population (age-adjusted, according to Adult Treatment Plan III (ATP III) standards) using NHANES III data [35,36]. Metabolic syndrome was prevalent in 23% of AAs according to ATP III standards and in 28% of AAs according to World Health Organization (WHO) standards [35].

Three studies explicitly assessed the determinants of diabetes and related health indicators among AAs. In one study, age at immigration, duration in the US, activity in Arab organizations, and consumption of Arabic food were all shown to be associated with increased risk for dysglycemia after adjusting for age and BMI [37]. Another study showed that length of time in the US was negatively associated with diabetes prevalence [14]. A different study showed that determinants of diabetes among AAs also included older age, higher BMI, higher diastolic and systolic blood pressures, and higher total cholesterol and triglyceride counts [32]. The determinants of non-insulin dependant diabetes mellitus (NIDDM) among AAs, aside from acculturation, are similar to those among the general public [38].

Berlie and colleagues [39] assessed adherence to ADA guidelines among 53 AAs with diagnoses of Type II diabetes. AAs had better lipid control but worse blood pressure control than the general public. Pharmacotherapy among AAs was less aggressive than recommended by the ADA.

### Psychological Well-being

Three studies have assessed the determinants of AA mental health in the US and three have assessed the mental health of Iraqi refugees specifically. A study among AAs in northcentral Florida showed that self-reports of recent discrimination were positively correlated with psychological distress and negatively correlated with self-esteem and environmental mastery [40]. A separate study among AAs from 19 states and the District of Columbia (DC) showed that among Christian AAs, less integration and more marginalization were associated with lower family dysfunction. Greater family dysfunction along with greater Arab religious/family values and acculturative stress were predictors of depression. However, in the same study, among Muslim AAs, less Arab religious/family values, less religiosity and greater marginalization were associated with family dysfunction. Greater family dysfunction and less religiosity were associated with depression [11]. A third study concerning the psychological well-being of AA elders showed that immigrant status was associated with lower life satisfaction and more frequent feelings of depression [41]. Perceived discrimination and acculturative stress have also been shown to be associated with risk for mental disorder among other ethnic and racial minorities in the US [42-45].

Three studies assessed the mental health of Iraqi refugees. In a chart review of 375 AA mental health clinic patients, Iraqi patients were more likely to be diagnosed with post-traumatic stress disorder (PTSD), more likely to have physical complaints, and more likely to have full remission after care than other AA patients [46]. A different study found that the mean number of medical complaints among those Iraqi refugees with PTSD was 10.4, 9.4 among those with depression, 8.7 among those with other disorders, and 10.6 among those with unknown illness [47]. The third study, among 32 Iraqi Gulf war refugees, found that 59% of respondents met the threshold for PTSD, which was associated with specific distress associated with pain before war [48].

### Cancer

We are aware of only one study that was specifically concerned with cancer among AAs. Schwartz and colleagues [49] calculated age-adjusted AA proportional incidence ratios of site-specific cancers from all cancer cases in metropolitan Detroit between 1973–2002. AAs had 36% greater proportions of liver cancer, 44% greater thyroid cancer, 29% greater leukemia, 28% greater brain, 25% greater kidney, and 24% greater bladder cancers compared to non-Arab whites. They had 25% less skin melanoma, 27% less esophagus and 20% less oral cavity cancers than non-Arab whites.

### Women and Children's Health

Two studies assessed the prevalence and two assessed the determinants of adverse birth outcomes among AA women. AAs are at lower risk for adverse birth outcomes than the general US population. One study considered all births in Michigan between 1993–2002. Among this sample, the prevalence of preterm birth (PTB) among women of Arab ancestry was 8%, and the prevalence of PTB among foreign-born AA women was significantly lower than that among non-Hispanic white women (9%) [50]. Another study found that the prevalence of low birth weight (LBW) among a sample of 823 AA mothers enrolled in the Women, Infants, and Children program in Dearborn, Michigan (MI) was 5%, which was lower than the prevalence of LBW in both Michigan (7%) and the US (8%) [21].

Two studies assessed the determinants of adverse birth outcomes among AA women. According to El Reda and colleagues [50], determinants of PTB among native-born AA women include pregnancy-related hypertension and lack of prenatal care visits. In the same study, determinants of PTB among foreign-born AA women were use of Medicaid, pregnancy-related hypertension, and diabetes. Those among native-born whites were age greater than 35, education below high school, lack of prenatal care visits, male gendered infant, tobacco use during pregnancy, chronic hypertension, pregnancy-related hypertension, and diabetes [50].

In a specific analysis concerning risk for adverse birth outcomes in the context of maternal stress, Lauderdale [51] assessed the effects of the terrorist attacks of September 11, 2001 on risk for adverse birth outcomes among AAs in California. Risk for LBW (OR = 1.3) and PTB (OR = 1.5) increased among Arab-named mothers in the six months following the terrorist attacks of September 11, 2001 as compared to the same six-month period in the year prior. If the infant's name was ethnically distinctive, relative risk for LBW among AAs was 2.3. Lauderdale found no change in the birth outcomes of non-Arab named women following the attacks of September 11, 2001 as compared to before the attacks.

### Health and Illness Psychology

The literature about the health and illness psychology of AAs is concerned with self-reported health and activity limitations, health care needs, HIV/AIDS knowledge and attitudes, and attitudes toward domestic abuse among AAs.

Read and colleagues [52] assessed comparative self-rated health and activity limitations between Arab immigrants and US-born whites. They found that Arab immigrants do not differ from US-born whites in self-rated health and are less likely to report limitations in activity after adjustment for demographic and cultural factors. Arab immigrants who are citizens report worse health while their peers who are not officially American do not significantly differ from the general population after controlling for duration of US residency.

In a subjective health care needs assessment among 15 men and 32 women in California, Laffrey and colleagues [53] found that the five most prevalent health concerns in the AA community were upper respiratory infection (36%), cardiovascular disease/hypertension (23%), emotional problems (17%), diabetes (15%), and social/family stress (13%).

Kulwicki and Cass [54] assessed the knowledge and attitudes of AAs about HIV/AIDS. AAs were generally less knowledgeable about HIV/AIDS transmission and had more misconceptions about the disease than the general public in the US based on NHIS data [55].

One study assessed attitudes toward domestic abuse among AAs [56]. Fifty-eight percent of women and 59% of men approved of a man slapping his wife if she hit him first during an argument. If a man learned of his wife's infidelity, 48% of women and 23% of men approved of the man slapping her. Eighteen percent of women believed a man could kill his wife if she was unfaithful.

### Miscellaneous Reports

Miscellaneous reports about the health of AAs were concerned with proportional cause-specific mortality, asthma, cystic fibrosis (CF) carrier frequency, trace element profiles, and dermatologic diseases.

Nasseri [57] explored proportional mortality ratios (PMR) among AAs in California for several common causes of mortality. PMRs for coronary heart disease (PMR = 1.3), suicide (PMR = 2.0), and diabetes (PMR = 1.9) among AA men were significantly elevated compared to non-Hispanic white men. Those for chronic obstructive pulmonary disorder (COPD) (PMR = 0.6) and HIV/AIDS (PMR = 0.5) were significantly lower among AA men than among non-Hispanic white men. PMRs for coronary heart disease (PMR = 1.3) and diabetes (PMR = 2.0) among AA women were significantly elevated compared to non-Hispanic white women and those for COPD (PMR = 0.2) and homicide (PMR = 0.5) among AA women were significantly lower compared to non-Hispanic white women [57].

The prevalence and correlates of asthma among AAs were studied by Johnson and colleagues [58]. They found that the prevalence of asthma among AAs was lower than among the general population in Michigan and the US. Twelve percent of the adult AA population was identified as being at high risk for asthma, while 8% had been diagnosed with asthma. This compares to 10% in Michigan as well as nationwide that had been diagnosed with asthma during their lifetime in a published report [59]. Age, acculturation, and socioeconomic status (SES) were predictive of asthma prevalence [59].

Wei and colleagues [60] studied CF carrier frequency among AAs and found that 1/115 of 805 AA patients undergoing preconception CF career screening in Detroit, MI were carriers. The observed CF carrier frequency among AAs was lower than frequencies published for African (1/61) and Hispanic Americans (1/58) in the US [61,62].

Trace element profiles in the toenails of 259 AA adults and children living in metropolitan Detroit, MI were also assessed by Slotnick and colleagues [63]. Compared to published means [64-67] Arab-Americans have lower mean values of the essential elements copper, manganese, and selenium and higher values of the nonessential elements aluminum, cadmium, and nickel.

El-Essawi and colleagues [68] assessed the prevalence of common dermatologic conditions among AAs. They found that the five most prevalent dermatologic conditions among AAs were acne (38%), eczema/dermatitis (26%), warts (20%), fungal skin infections (20%), and melasma (15%). Acne and eczema were also among the most prevalent dermatologic conditions among African-Americans [69] and Hispanic-Americans [70].

## Discussion

In this systematic review about the health of AAs in the US, we found that 1) there is little consensus about the relative burdens of CVD or diabetes among AAs compared to other groups, however the best available evidence suggests that the prevalence of hypertension among AAs is between 13–20% and the prevalence of diabetes is between 5–33%; 2) there is limited information about the prevalence of cancers or common mental disorders in this group; 3) there is evidence suggesting that AAs have lower prevalence of adverse birth outcomes and adolescent tobacco use compared to the general US population and to majority groups in the US; 4) the determinants of common morbidities among AAs are similar to those within the general population, although there is evidence that acculturation, immigration, and discrimination-associated stress are involved in the etiologies of CVD, diabetes, mental illness, and/or adverse birth outcomes among AAs.

There are several methodological limitations to the extant literature that challenge our current understanding of the health of AAs in the US and hinder the systematic comparison of the health of AAs to the health of other ethnic groups in the US. We will discuss each of these limitations in turn aiming to describe how these limitations influence our interpretation of the literature.

First, the central limitation to our understanding of the health of AAs in the US is the relative paucity of published studies concerning the health of this ethnic group. Our search of the literature yielded only 34 studies. This challenges our inference about the health of AAs in the US in several ways. First, there is little consensus about the prevalence and correlates of major morbidities such as diabetes and CVD among AAs relative to other ethnic groups. Because these diseases account for much of the chronic disease burden in the US, understanding the prevalence and correlates of these diseases among AAs is a primary concern. Second, there is little to no research about the prevalence and correlates of several important health indicators, such as all-cause mortality, common mental disorders, site-specific cancers, HIV/AIDS and other infectious diseases, and chronic pain conditions. Third, the literature about AA health has yielded few studies explicitly concerned with the health effects of potentially important exposures, such as immigration, acculturation, and discrimination among AAs. For example, as an ethnic group, AAs have been systematically targeted for discrimination–more so since the terrorist attacks of September 11, 2001 and the subsequent wars in Afghanistan and Iraq [4,5]. However, there are only two studies of which we are aware that explicitly explore the relation between discrimination and health among AAs. One study considered the effects of post-September 11, 2001 discrimination-associated stress on AA birth outcomes, and the other considered the effects of discrimination on AA mental health. Both studies found that discrimination was either indirectly or directly associated with worse health among AAs [40,51]. However, because the current literature about the health effects of discrimination among AAs is limited to two studies, the effects of discrimination cannot be generalized to health indicators that have not been explicitly associated with this exposure. Similarly, there are few studies that systematically assess the effects of immigration and acculturation on AA health.

The second methodological limitation to our understanding of AA health is the paucity of prospective studies that include data about AAs. Twenty-seven out of 34 studies that assessed the health of AAs in the US used cross-sectional analyses. The remaining 7 studies used either retrospective cohort analyses or medical chart reviews. We did not identify any studies that prospectively assessed the health of Arab-Americans in the US. Prospective cohort studies about the health of AAs in the US would be important for several reasons. First, without the use of prospective studies it is impossible to establish population-based incidence rates for health indicators among AAs. Second, prospective studies would allow inference about the causal and temporal mechanisms that underlie associations between Arab ethnicity and health indicators. Third, prospective studies are needed to help understand whether some of the observed associations between social exposures such as immigration, acculturation, and discrimination and health play different roles across the life course [71,72]. A corollary to this limitation is that there is essentially no literature about the health of Arab immigrants to the US at baseline. We are not aware of any studies that assess the health of new arrivals to the US from Arab countries and assess subsequent changes in health post-immigration.

A third limitation is that many of the extant studies about the health of AAs use place of birth to demarcate Arab ethnicity. The use of place of birth as a marker for ethnicity unfortunately confounds ethnicity with migrant status which presents particular challenges. Migrant status in and of itself may be a determinant of AA health, positively or negatively [73-81]. In addition, there are likely factors that are associated with the health of migrants that are not relevant to the US-born AA population. For example, it has been suggested that acculturation is associated with both better [37] and worse [11,54,56] health indicators among AAs. The use of place of birth as a marker for ethnicity in a large portion of the literature about the health of AAs precludes the generalizability of study findings to native-born AAs.

Fourth, many of the extant studies concerning the health of AA have used convenience samples in one area with a high AA concentration. Twenty-six of the 34 (76%) studies reviewed above sampled AAs in Michigan; 24 of those 26 in Michigan sampled AAs in the southeast part of the state, which includes metropolitan Detroit and Dearborn, MI. Because of its high concentration of AAs, metropolitan Detroit is an attractive study locale for investigators interested in the health of AAs. However, it is possible that health indicators among AAs in Michigan are not representative of those among the US population of AAs. For example, although national data show that AAs are on average more affluent and educated than the general US population [3], SES indicators in health studies among AAs in Michigan are generally lower than those among the general population of the state [20,31,35,50,52]. Alternatively, it has been shown that ethnic minorities who live in localities with high concentrations of members of their ethnic group are protected against adverse health outcomes [82-85]. Because southeast Michigan is home to the highest concentration of AAs in the US, it is possible that the health of AAs in southeast Michigan differs meaningfully from the health of AAs in other localities in the US.

There are several cautions that should be noted when interpreting the findings of this review. First, we organized our findings around eight morbidity clusters. It is important to note that this organization was likely influenced by the search terms used in the article search methodology. Second, because our inclusion criteria restricted the articles reviewed herein to those published in peer-reviewed journals, there may be publication bias regarding the articles discussed in this review. Therefore, our findings may not accurately reflect current knowledge about Arab-American health in the US. Third, while several of the topics discussed in articles reviewed in the "Miscellaneous Reports" portion of the results may represent defined morbidity clusters in their own right, there was not sufficient published research to establish any of them as such with regard to the health of AAs living in the US.

## Conclusion

We suggest that five directions for future research emerge from this review. First, investigators interested in the health of AAs or in understanding the health effects of social exposures such as immigration, acculturation and/or discrimination might consider undertaking prospective cohort studies of the health of AAs in the US. Second, because the majority of studies published concerning the health of AAs in the US are set in southeast Michigan, investigators interested in the health of this ethnic group could consider studies using national and statewide datasets or studying the health of AAs in different localities. Third, devising better and more efficient methods of determining Arab ethnicity, such as validated name algorithms [86,87], may help provide investigators with tools to identify AAs that may be useful for population-based studies. Fourth, state and local health departments concerned with the health of AAs living within their territories might consider collecting data on Arab ancestry or ethnicity to improve health and demographic research about this population. Fifth, little is known about the comparative health of AAs in the US and Arabs in Arab countries. Future work may fruitfully assess the health of AAs on immigration to the US and how the post-immigration environment influences subsequent health in this group and accounts for differences in health indicators between AAs and other ethnic groups in the US or between AAs in the US and Arabs worldwide.

## Abbreviations

AAs: Arab-Americans; AA: Arab-American; US: United States; MI: Michigan; NHANES III: Third National Health and Nutrition Examination Survey; NHIS: National Health Interview Survey; PTSD: Post-traumatic stress disorder; CVD: Cardiovascular disease; HDL: High density lipoprotein; BMI: Body mass index; ATP III: Adult Treatment Plan III; WHO: World Health Organization; ADA: American Diabetes Association; A1c: Hemoglobin A1c; FL: Florida; PIR: Proportional incidence ratio; DC: District of Columbia; PTB: Preterm Birth; LBW: Low birth weight; HIV/AIDS: Human immunodeficiency virus/acquired immunodeficiency syndrome; PMR: Proportional mortality ratio; COPD: Chronic obstructive pulmonary disorder; SES: Socioeconomic status; CF: Cystic fibrosis; NIDDM: non-insulin dependant diabetes mellitus.

## Competing interests

The authors declare that they have no competing interests.

## Authors' contributions

AMES conceived the study, carried out the search, and wrote the manuscript. SG edited the manuscript and participated in its design and coordination. All authors read and approved the final manuscript.

## Pre-publication history

The pre-publication history for this paper can be accessed here:



## Supplementary Material

Additional file 1 **El-Sayed and Galea review search strategy**. Details about the search strategy employed during the literature search and results thereof. Click here for file

Additional File 2 **Arab-American Health Table S1**. Details about each of the studies. Click here for file
