# Supplementary material for: The health of Arab-Americans living in the United States: a systematic review of the literature
Source: BMC Public Health. 2009 Jul 30;9:272. doi: 10.1186/1471-2458-9-272 (PMC2728720; doi:10.1186/1471-2458-9-272)
Supplement: Additional file 1 — El-Sayed and Galea review search strategy. Details about the search strategy employed during the literature search and results thereof. [file 1471-2458-9-272-S1.pdf]

"The health of Arab-Americans living in the United States: a systematic review of the literature"

1. All search terms were queried into the MEDLINE, Web of Science, BIOSYS previews, and Current Content Connect databases using the Web of Knowledge interface:

1) ((Arab OR "Middle*Eastern" AND America*) AND (tobacco OR smok* OR water*pipe))

2) ((Arab OR "Middle*Eastern" AND America*) AND ("cardiovascular disease" OR "heart disease" OR infarct* OR heart OR MI OR CVD OR atherosclero* OR stroke OR ischemi*))

3) ((Arab AND America*) OR (Chaldean AND America*) AND (Cancer OR neoplas* OR Tumor OR leukemia OR lymphoma))

4) ((Arab AND America*) OR (Chaldean AND America*) AND (Diabet* OR glucose OR metaboli*))

5) ((Arab AND America*) OR (Chaldean AND America*) AND (matern* OR pregnan* OR ped* OR preterm OR birth OR abuse))

6) ((Arab AND America*) OR (Chaldean AND America*) AND (depress* OR anxiety OR PTSD OR post*trauma* OR substance OR mood OR schizophren* OR mental OR psych*))

2. This search process yielded a total of 1336 studies of which 88 were kept. From these studies, 54 were removed because they were either meeting abstracts or were not empiric in design (i.e., commentaries or discussion pieces).

3. The references of each of the 34 included studies were then searched for studies concerned with the health of Arab-Americans in the United States that may not have been detected via our initial search strategy. No such studies were found.
